# Realizing the Potential of Computer-Assisted Surgery by Embedding Digital Twin Technology

**DOI:** 10.2196/35138

**Published:** 2022-11-08

**Authors:** Jiaxin Qin, Jian Wu

**Affiliations:** 1 Institute of Biomedical Engineering Shenzhen International Graduate School Tsinghua University Shenzhen China

**Keywords:** computer-assisted surgery, digital twin, virtual space, surgical navigation, remote surgery

## Abstract

The value of virtual world and digital phenotyping has been demonstrated in several fields, and their applications in the field of surgery are worthy of attention and exploration. This viewpoint describes the necessity and approach to understanding the deeper potential of computer-assisted surgery through interaction and symbiosis between virtual and real spaces. We propose to embed digital twin technology into all aspects of computer-assisted surgery rather than just the surgical object and further apply it to the whole process from patient treatment to recovery. A more personalized, precise, and predictable surgery is our vision.

## Introduction

As is well known, computer-assisted surgery (CAS) has been widely used in the field of surgery. It enables the precise positioning and visualization of the patient’s deconstructive structures and surgical instruments by integrating medical imaging, spatial positioning technology, and various computer technologies. Therefore, with CAS, the surgeon can plan the surgical procedure in a precise manner before the operation, such as the access point, implant selection, and placement [1]. However, intraoperative planning cannot be implemented accurately during surgery due to intraoperative disturbances such as respiratory movements and tissue deformation. Moreover, CAS is still lacking in postoperative prediction, prevention of surgical complications, and postoperative evaluation.

Digital twin (DT) is a concept or technology that refers to create a multiphysical, multiscale, and high-fidelity virtual representation of physical entity in the virtual space. We call this virtual representation the DT of its corresponding physical entity. The virtual representation can be dynamically updated when the physical entities change, enabling real-time mapping from physical space to virtual space. DT originates in the industry [2] and is now used in many fields such as precision health [3], manufacturing, construction, product design, and weather prediction [4]. Considering the features of CAS and DT, we propose to bring DT to CAS and explain why and how DT can be used to enhance the application of CAS in all its phases, especially the potential value in remote surgery when combining CAS with DT.

## Bringing DT to CAS

**Figure 1:**
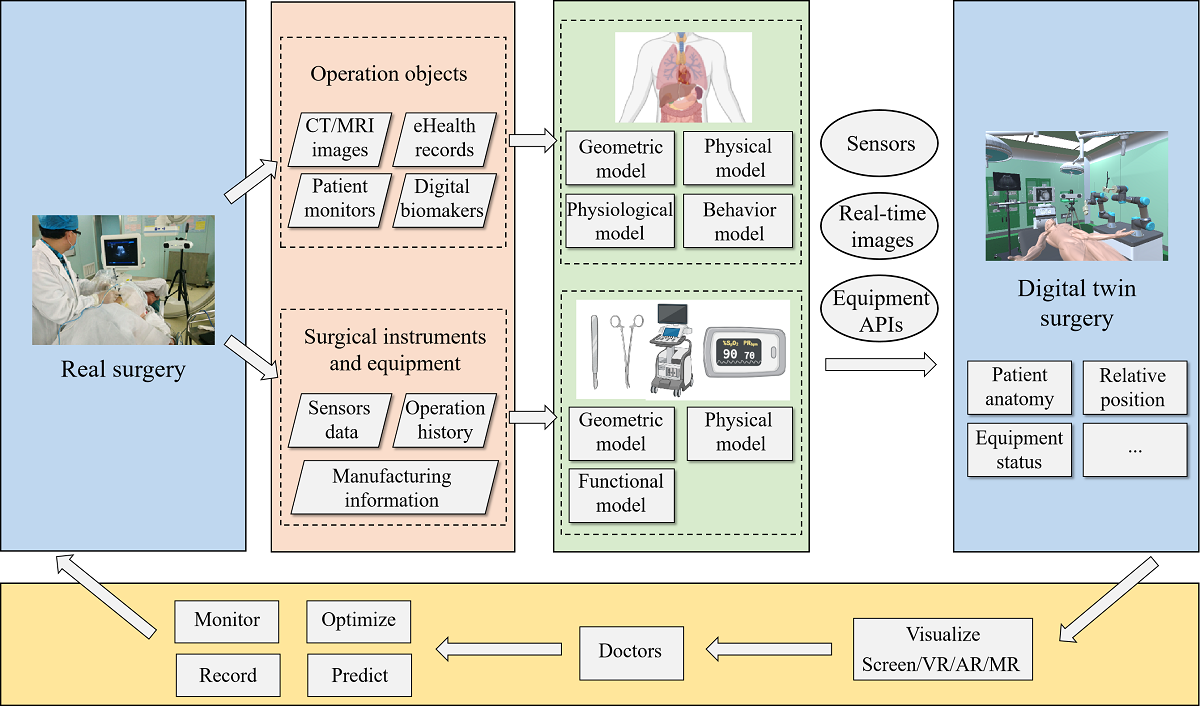
Digital twin–based computer-assisted surgery (CAS) solution. With the support of digital twin surgery, we can perform monitoring, optimization, recording, and prediction for the real surgery. API: application programming interface; AR: augmented reality; CT: computed tomography; MR: mixed reality; MRI: magnetic resonance imaging; VR: virtual reality.

Figure 1 illustrates our proposed DT-based CAS solution. We collect data related to surgical objects, surgical instruments, and medical devices in the preoperative period, and use these data to build their multiphysical, multiscale, and high-fidelity DT models in virtual space by mathematical simulation and modeling [5]. DT models can dynamically simulate a wide range of properties of the patient’s tissues and organs, such as geometric, physical, physiological, and behavioral properties. Therefore, preoperative planning, surgical simulation, and postoperative prediction based on these models will be more accurate and comprehensive.

Information, as well as the changes of information from surgical instruments and the patient (such as anatomical structure, position, posture, etc), are acquired during surgery using real-time imaging technologies and a variety of sensors. Due to the high requirements for real-time performance, it is much more difficult to obtain whole information intraoperatively compared to preoperatively. For example, the clarity of the patient’s anatomical structure obtained by ultrasound imaging is much less than that of preoperative CT (computed tomography) and MRI (magnetic resonance imaging). However, we can quickly capture local feature information from ultrasound images through advanced methods such as deep learning [6]. Combining this local information with the preoperative DT models, which already have physical, physiological, and behavioral characteristics, it is possible to obtain the overall dynamic changes of the patient’s anatomical structure. In this way, intraoperative changes can be evaluated in real time, and all the information in the virtual space can be visualized when using extended reality (virtual reality, augmented reality, and mixed reality), and it also provides the surgeon with real-time, accurate surgical navigation.

Sensors can collect a variety of data that are needed to update the DT during the procedure. Commonly used sensors are physiological, mechanical, and position sensors. Among them, position sensors play a key role in positioning and tracking in surgical navigation and can help achieve a baseline position mapping relationship between the real surgical space and the DT surgical space. Optical sensors have higher accuracy and real-time performance compared with other position sensors. They are able to collect position and posture data of the optical marker in real time in the form of quaternions or transformation matrix. We usually fix the optical marker on the object of interest. For example, we can fix the optical marker on the ultrasound probe before the procedure and obtain the conversion between the optical marker coordinate system and the ultrasound image coordinate system by ultrasound probe calibration [7]. Intraoperatively, we can not only collect real-time position and posture information of the ultrasound probe in real time but also obtain the spatial position of any point displayed on the ultrasound image.

An application programming interface is a software intermediary that can create data links between different devices. We can use application programming interfaces of medical devices to obtain operational data in real-time and update the DTs. Through these DTs, we can monitor, control, and manage medical devices in a uniform way.

While the surgery is being performed in real space, a digital record of the surgery is updated simultaneously in virtual space, which we call the process twin of the whole surgery process. This record can be used for postoperative evaluation of the patient, including the evaluation of the actual surgery, the surgical procedure, the choice of strategy, and so on. It can also act as an important reference for postoperative follow-up.

## Potential in Remote Surgery

It is the establishment of an accurate match between the real world and the virtual space that enables effective remote surgery on real patients using DTs. The surgeon can obtain the real-time situation of the surgery at the remote end by the dynamically updated DTs and can use a haptic device to remotely control the robotic arm to perform the surgery [8] through high-speed, low-latency communication technologies such as 5G. Since the DTs contain rich information about the surgical object, the surgeon can obtain important feedback information [9] at the remote end through the haptic device without any other sensors. For each surgical device, it can also be monitored and controlled remotely through their DTs in virtual space.

## Data Processing and Security

Data-driven approach is one of the core approaches to implement DT. The process of dynamically updating the DTs intraoperatively requires a large amount of data computation and interchange. By using cloud computing technology [10], hardware costs can be effectively reduced. Medical Cyber Physical Systems are the networked health care integration of medical devices [11]. They provide a superior way to capture, store, and securely access large amounts of medical data. In the future, its development may provide important data support for the application of DT [12]. Additional attention needs to be paid to the fact that the collection of private health data on human individuals may raise complex ethical issues [13]. Thus, data security should be carefully considered for the storage and retrieval of operation data, and data process encryption should be embodied in DT.

## Discussion

Bringing DT to CAS is to monitor, optimize, record, and predict the surgical process in the real space by creating a virtual twin surgical space (including the DTs of the surgical object, surgical instruments, and medical equipment). In this way, it can leverage and integrate data from the entire surgical phase and is applied to the patient from treatment to recovery. This also determines the higher level of complexity of the DT systems. Different types of data from multiple devices need to be integrated within the same system and ensure the system’s stable operation. The real-time dynamic response of the DTs requires high data transmission speed and network speed, especially in the remote surgery. In addition, the simulation of complex physiological signals of biological tissues is still a challenge that needs to be faced if a more detailed patient model is desired. In the future, the virtual twin space can be used as a carrier to establish an integrated, digital surgical process management system and to form a new clinical implementation system. Under such a system, surgical treatment will be more personalized, precise, and predictable.

## Abbreviations

CAS: computer-assisted surgery
CT: computed tomography
DT: digital twin
MRI: magnetic resonance imaging
